# Synthetic circuit designs for earth terraformation

**DOI:** 10.1186/s13062-015-0064-7

**Published:** 2015-07-18

**Authors:** Ricard V. Solé, Raúl Montañez, Salva Duran-Nebreda

**Affiliations:** Institució Catalana per a la Recerca i Estudis Avançats-Complex Systems Lab, Universitat Pompeu Fabra, 08003 Barcelona, Spain; Institut de Biologia Evolutiva, (CSIC-Universitat Pompeu Fabra), Passeig Marítim de la Barceloneta 37, 08003 Barcelona, Spain; Santa Fe Institute, 1399 Hyde Park road, Santa Fe, NM 87501 USA

**Keywords:** Synthetic biology, Ecological engineering, Climate change, Catastrophic shifts, Mutualism

## Abstract

**Background:**

Mounting evidence indicates that our planet might experience runaway effects associated to rising temperatures and ecosystem overexploitation, leading to catastrophic shifts on short time scales. Remediation scenarios capable of counterbalancing these effects involve geoengineering, sustainable practices and carbon sequestration, among others. None of these scenarios seems powerful enough to achieve the desired restoration of safe boundaries.

**Presentation of the hypothesis:**

We hypothesize that synthetic organisms with the appropriate engineering design could be used to safely prevent declines in some stressed ecosystems and help improving carbon sequestration. Such schemes would include engineering mutualistic dependencies preventing undesired evolutionary processes. We hypothesize that some particular design principles introduce unescapable constraints to the engineered organisms that act as effective firewalls.

**Testing the hypothesis:**

Testing this designed organisms can be achieved by using controlled bioreactor models, with single and heterogeneous populations, and accurate computational models including different scales (from genetic constructs and metabolic pathways to population dynamics).

**Implications of the hypothesis:**

Our hypothesis heads towards a future anthropogenic action that should effectively act as Terraforming processes. It also implies a major challenge in the existing biosafety policies, since we suggest release of modified organisms as potentially necessary strategy for success.

**Reviewers:**

This article was reviewed by This article was reviewed by Eugene V. Koonin, Tom Ellis (nominated by Purificación Lopez-Garcia) and Eörs Szathmary.

“*The future cannot be predicted, but futures can be invented*” Dennis Gabor

## Background

Climate change, along with a rapid depletion of natural resources and biodiversity declines is driving the biosphere towards unstable states. Widespread evidence indicates that increasing rise of average temperatures is leading to local, regional and global modifications of extant habitats, seriously endangering the future of our planet [3, 25]. Given the large scale of the problem, suggested scenarios based on human intervention might fail to properly address the ongoing changes. Additionally, the time evolution of these changes can rapidly accelerate due to runaway effects associated to the nonlinear nature of these phenomena. In other words, current continuous changes might end up in so called *catastrophic shifts* [36, 56, 57]. Are we going to be capable to avoid them?

Future scientific and engineer efforts toward a better understanding of these changes have to come up, in parallel, with potential remediation scenarios to ameliorate and even stop the current trends. In such a way, different strategies involving mitigation [17] geoengineering [10, 59, 67] or adaptation [20] have been proposed.

Mitigation implies measures that slowdown ongoing emission rates or provide ways for limiting emissions. Geoengineering, in the other hand, explicitly requires directed changes that have been questioned due to staggering costs, unknown outcomes and limited impact (particularly in relation with CO_2_), which make unclear their potential for counterbalancing current trends [32, 53, 59]. Adaptation scenarios place us in a future world where we will need to cope with new environmental and economic constraints.

None of these suggested solutions might be a definite solution, but clearly the price for inaction will be much larger than any of the previous possibilities.

It has been recently suggested that an alternative possibility would involve actively acting on the biosphere through the use of synthetic biology, from an artistic [Alexandra] and scientific [64] point of view. This approach could be used, among other things, as a way to curtail the accumulation of greenhouse gases, enhance nitrogen fixation or slow down degradation in arid and semiarid ecosystems. The key point of this proposal is that engineering living systems allows to reach large scales thanks to the intrinsic growth of the synthetic organisms. This makes a big difference in relation to standard engineering schemes, where artifacts need to be fully constructed from scratch. Instead, once a designed population is released, appropriate conditions will allow the living machines to make copies of themselves and expand to the desired spatial and temporal scales, **or even spread an engineered device** [19].

This approach, which is an effective way of “Terraforming” the biosphere, needs to consider potential scenarios that guarantee an efficient result as well as a confined evolutionary potential. In this context, target habitats for designed organisms should be chosen as an additional, ecological-level containment strategy. Moreover, limits to the impact of synthetic organisms can be obtained using ecological interactions that are based on either cooperative loops or habitat constraints that are especially well met by different classes of anthropogenic-modified scenarios. Designed microbes capable of functioning only under specific conditions have been constructed and strategies to incorporate genetic safeguards explored [51, 42, 39]. One avenue, to be used in biomedical applications, is to force the need for xenobiotic (unnatural) molecules that need to be supplied along with the genetically modified bacteria [39]. In this paper we consider four possible engineering motifs that can cope with these two constraints. We do not consider explicit case studies (i. e. detailed genetic constructs or designed organisms) but instead the logic design schemes.

## Presentation of the hypothesis

The obvious criticism to the scenario presented in [69] has to do with the unknown consequences of ecological and evolutionary dynamics on the engineered ecosystems. Actually, it can be argued that well known cases of exotic species introduced in some ecosystems caused large-scale disaster [54, 62]. The list includes the introduction of different kinds of species into a novel habitat where they benefited from a higher efficiency to exploit available resources. This situation corresponds (at least transiently) to a population positive feedback loop that involves an accelerated expansion (typically exponential in its first phase). Is there a rational strategy that can minimize the impact of an engineered species? One way of preventing undesired explosive growth is engineering a strong ecological link between organisms, whose interdependence turns into a limitation for their spread. To do this, a selected organism present in the target habitat has to be modified in order to implement the aforementioned ecological dependency. That would result in population dynamical processes preventing unbounded growth of the modified organism. Moreover, using the appropriate context, strong habitat constraints can act in synergy as ecological firewalls.

Here we suggest that two main avenues can be followed. One is engineering mutualistic relationships with resident organisms through the modification of already extant organisms. Recent experimental studies indicate that such designed mutualistic link can be created by artificially forcing a strong metabolic dependence and also with the help of genetic engineering [22, 33]. These studies have shown that the end product can be a physically interacting, stable pairwise relationship [43]. Another possibility is to use a modified organism that grows on a given waste-related substrate that can be preferentially (or exclusively) used, and may be degraded, by the synthetic organism. Such substrate can be plastic garbage, sewage and other sources of human-created waste. Additionally, some special habitats might be ideal to grow strains of engineered organism capable of performing a given functional task and unable to survive outside their restricted environment. In the next section, we consider a list of candidate engineering designs (and their variants) that could fit the previous description. We will define their basic logic and outline potential scenarios for their implementation, as well as potential drawbacks.

### Synthetic terraformation motifs

In this paper we introduce four potential bioengineering schemes. Hereafter, **H** and **SYN** indicate the target host and a synthetic microbe, respectively. What we consider host and synthetic microbe depends on specific situations tackled. Considering a semiarid ecosystem, a plant complementing an engineered auxotrophy in the **SYN** can be considered as a **H**. On this same environment, a **SYN** might been a auxotrophyc microorganism, obtained from some existing wild type strain (**WT**), engineered to be auxotrophyc and to produce some hygroscopic molecules able to retain water, closing in this way the mutualistic loop. Similarly, **R** is used to indicate some sort of resource, while **W** stands for water. The basic designs are intended to represent the logical organization of our proposed constructs, and not the specific genetic designs. For this reason, since they are introduced as logic graphs, we choose to call them *Terraformation motifs* (**TMs**) to indicate this logic nature. The first two motifs deal with the engineering of cooperative interactions, either directly or indirectly. The third incorporates a design principle grounded in a tight dependence of the engineered microbes with a specific class of available resource or physical support. The fourth, involves the use of an existing human-generated waste habitats, as the niche of engineered microbes, which will be controlled through some class of lethality outside their selective environmental niche.

### Engineered mutualism

**Fig. 1:**
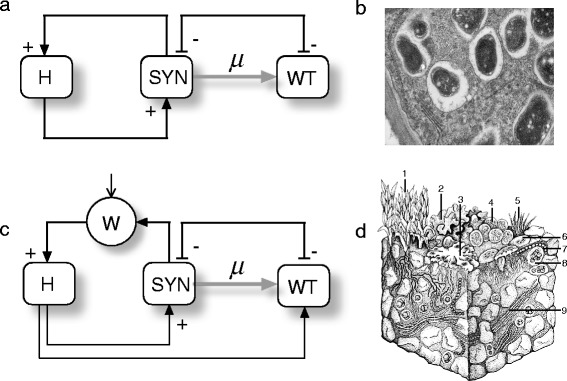
Terraformation motifs involving closed cooperation among players. Two main classes ofpotential engineered synthetic microbes (SYN) interacting with their hosts (H) are indicated. Assuming that theengineered species has been obtained from an existing one in the same environment, the wild type (hereindicated as WT) can be obtained from SYN if the engineered construct is lost by mutation (here indicated as afray arrow, and as a rate μ) As SYN and WT are in essence the same organisms, they compete for the sameresources. In (**a**) we display a logic diagram of positive interactions among both partners defining a mutualdependency. In (**b**) such cooperative interaction is mediated through some class of physical factor, such aswater (W). These two classes correspond, for example, to exclusive mutualistic interactions displayed by plantcells within root nodules (**c**) where nitrogen-fixing bacteria are physically embedded (image from http://en.wikipedia.org/wiki/Rootnodule). On the other hand, the need for survival under stressful conditions,as those common in arid ecosystems, makes water a major player and limiting resource. An engineeredmicrobe capable of improving moisture retention can have a very strong effect on the underlying plantspecies, expanding their populations. In soil crusts (**d**) a whole range of species exist, adapted to water-poorconditions (drawing adapted from Belnap et al 2001). Here we indicate (1) mosses (2,3) lichens, (4,5,7,9) cyanobacteria, (6) fungi and (8) green algae.

In this case, a **WT** organism, isolated from the selected environment, is transformed into a **SYN** by engineering a mutual dependency with a candidate **H**. Mutual dependency can be implemented in many different ways. A natural example to follow is the interdependent relationship of Rhizobium with legume plants (Fig.1c). In this system, Rhizobium fixes nitrogen from the air into ammonia, which acts as a natural fertilizer for the plants, while plant provide a protective and nutritive niche to bacterial growth. Engineered mutualism goes beyond nature, imposing dependencies that can improve the fitness of the system, but also becomes in a containment mechanism, since the system failure ends in the disappearance of the modified species. In Fig. 1a we display the **TM** associated to this approach. Here the host and the synthetic organism have been designed to enhance each other’s growth. Moreover, the synthetic species has been derived from an existing wild type strain and it can thus mutate, with high probability, into something similar to **WT**. This will be the case if the engineered part is not enough advantageous and instead becomes a burden for the microorganism. In this scenario, **SYN** will have to compete with the present, and more abundant, **WT** organisms. Several experimental approaches have shown that such mutualistic relationship can be enforced by co-evolving plants and bacteria under strong selection together with genetic engineering. Engineering mutualistic symbiosis is already a reality. Proper manipulations of free-living species allow to force them to become obligate mutualists. This includes synthetic cooperative strains [61] evolving a plant pathogen into a legume symbiont [22, 40], fungal-plant mycorrhizal symbiosis [33] yeast-alga and fungi-alga associations created through a forced environmental change [24] or by means of long-term selection experiments enforcing metabolic dependencies [23] among others.

### Indirect cooperation

Cooperation can also arise from an interfaced interaction where one of the species modifies the existing medium in such a way that the partner can thrive and create more growth opportunities for the first. One motif that can meet the canonical definition of indirect fit benefit can be a species of microbe that has been engineered to excrete a molecule capable of enhancing water retention in arid conditions (Fig. 1b). Again, a preexisting organism in the chosen context can be engineered in order to release some kind of hygroscopic molecule capable of enhancing water retention. Potential candidates would be engineered cyanobacteria that are known to produce extracellular polysaccharides [38, 46]. Enhanced production of these molecules by synthetic strains could easily improve dry land soils and yield. Soil crust in particular, covers most soil surface in desert and constitute a crucial regulator of soil respiration in dryland ecosystems [48]. Accordingly, strategies oriented to soil rehabilitation and carbon sequestration could be implemented through the engineering of soil crust [6, 7]. When we look into the soil crust consortia (Fig. 1d), we see a set of different cyanobacteria interacting with other organism [4, 5].

Following the previous idea, engineered cyanobacteria incorporated to the soil crust can increase water retention, promoting growth in other organisms of the consortia of which cyanobacteria take also benefit. A similar strategy can be applied in plants of arid ecosystem. Plants can improve their growth thus expanding their population and providing further opportunities for microbial populations also to grow. In arid and semiarid habitats, plants typically develop local interactions involving so called *facilitation*: the presence of neighboring plants favors the establishment of others and the preservation of a healthy soil [9]. Given the constraints imposed by water shortage and overgrazing, patchy distributions of plants are the common pattern [31, 52, 55, 63]. Mounting evidence suggests that the conditions allowing these ecosystems to survive and the nonlinear nature of facilitation implies the existence of breakpoints and catastrophes: once reduced water availability or grazing pressure cross a given threshold, a rapid transition to the desert state should be expected. Modified organisms, capable of building an equivalent indirect co-operative loop as outlined above, would increase facilitation easily. The increasing role of arid and semi-arid ecosystems as carbon sinks [49] makes them a especially relevant target for our terraformation proposal.

### “Function and die” design

**Fig. 2:**
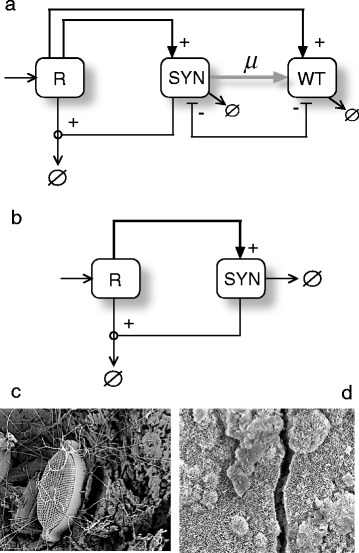
Function-and-die Terraformation motif. Here a given substrate R is being generated at a given rateand provides physical substrate to the synthetic population. The TM motif in (**a**) is based on the modification ofan extant species following the same criteria that described in figure 1, whereas in (**b**) we just assume that the engineered species has been improved to attach efficiently to the substrate. In both cases, the engineered species could perform a function while degrading the waste material ∅. Candidate examples are plastic ocean debris, where many species are known to live (**c**) or concrete cracks (**d**). figures (**c**) and (**d**) have been adapted from [11, 15], respectively.

An engineered microbe performing a given functionality (such as carbon sequestration) can be coupled to the degradation of a given resource, such as plastic garbage or other long-living byproducts of human activities. This scenario is strongly tied to the problem of bioremediation [11, 16]. Thereby, accumulated waste, consequence of anthropogenic actions, can be the resource (**R**) where a community of **SYN** performs a new ecological function like produce heavy metal chelation, capture CO_2_ or plastic aggregation. In Fig. 2a we consider a TM that follows our previous scheme (again, a synthetic strain is derived form an existing one). In this case, however, no mutualistic loop is at work. Instead, both SYN and WT would use the resource **R** and thus their populations depend on such potential for use it, which could be improved in the designed strain. A good candidate could be oceanic plastic garbage [2, 21], which is known that is colonized by many different species, including several microbial genera, such as *Vibrio* [70]. In this context, it is worth noting that, despite the rapid increase in plastic waste dumped in the ocean, the observed amount of plastic in open waters is much less than expected [35]. The former suggest (among other possibilities) that some microbial species capable to attach to plastic polymers are also degrading them. This observation indicates that evolutionary forces might have favored “plasticvorous” strains, which could be used as engineering targets. Additionally, different species, both prokaryotes and eukaryotes, are known to persist in plastic (Fig. 2c). Thereby, two possible approaches can be used that meet Fig. 2b. First, one organism able to break plastic can be engineered to attach or put together plastic, increasing the proportion of organisms in contact with the discarded plastic. Secondly, one of the organisms already attached to the plastic can be modified to produce enzymes able to break it. A different scenario that can be represented by our motif is provided by engineered bacteria that can be used to repair concrete cracks (Fig. 2d). The alkaline environment makes difficult for most species to thrive but some species can be used to this purpose [BacillaFilla]. Here the designed bacteria would enter, grow and replenish cracks with calcium carbonate until the task is finished. Several strategies have been used to this end and major improvements have been obtained [29, 50]. A major advantage of this problem is that anaerobic bacteria are not going to survive outside the crack and thus selection immediately acts once the task is finished. Once again, the right combination of genetic design and ecological constraints create a powerful safeguard against undesired evolution.

### Sewage synthetic microbiome

**Fig. 3:**
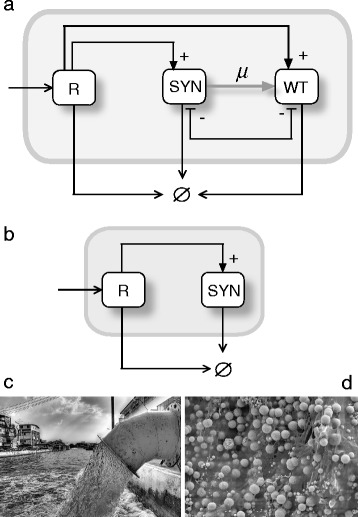
Sewage-based terraformation motif. In (**a**) we consider a situation where an artificial environment (grey box) is created as a byproduct of human activities, i.e. wastewater in sewer, and one extant organism is engineered. A simpler alternative (**b**) does not require engineering of extant species since it is a completely artifactual ecosystem and its preservation is not required. Our two strains are both sustained by available resource R and physical conditions (grey box) while they are growing there, but at some point all of them are removed (burned or released) at a given rate. A typical scenario would be sewage-related infrastructures (**c**) where a rich microbial community (**d**) is known to exist.

Urban cities are the largest human structures and as such they also incorporate massive infrastructures associated to treatment of waste as an end part of their metabolism. Sewage systems and landfills offer a especially interesting opportunity to apply our terraforming approach. It is known that sewage systems involve their own microbiome [44] and that some evolved microbes are currently causing damage to the concrete [45]. The existing sewage and urban microbiomes provide a rich repertoire of candidate species, although we just start to grasp their richness [1]. On the other hand, the sewage-based scenario is especially useful in our context, since microbes are eventually removed once they reach the open sea and their niches are disrupted by changes in osmolarity, pH or resources availability. If the same basic scheme is used, namely engineering an existing species, the TM can be summarized in Fig. 3a. Here a constant removal of both wastewaters and microbes is represented by the arrows ending as − > Ø. Sewage constitute one paradigmatic example of novel ecosystem created by humans to satisfy human necessities (Fig. 3c). Considering this artifactuality, to preserve the existing species of microbes might be a trivial effort, thus making unnecessary to engineer a wild type bacteria already present in the sewage. Then, sewage TM can be reduced to Fig. 3b. It also worth to note that foreign organism have to be able to compete with the already present sewage microbiome (Fig. 3d), being thereby relevant to assess the potential dynamic responses of the engineered ecosystems. An interesting connection between these potential engineered strains and the gut microbiome has been pointed in [64]. The later defines an enormously rich microbial ecosystem that has coevolved with our species through our long evolutionary history. Ongoing biomedical research starts to be oriented towards intervening in the microbiome by means of both drugs but also microbial strains that might act like exotic invaders aimed to restore lost functionalities [12, 13, 26, 47].

## Testing our hypothesis

History shows us that modification of ecosystems needs to be done carefully [30]. In consequence, the presented hypothesis has to be tested as deeply as be possible. We would test our hypothesis using three different approaches, *in silico* (applying computational models), at microscale (Testing the stability of the functions engineered into SYN and the population dynamics in bioreactors) and self-contained experimental system, which is larger than microbial cultivation systems but smaller than natural ecosystems (mesoscale) releasing SYN in complete ecosystems under controlled conditions. Computational models have to involve analysis of the genetic circuit implemented in synthetic organism and also of the population dynamics resulting of the release of the SYN in a selected ecosystem. Fortunately, five decades of work in systems ecology give us the required tools for that purpose [8, 34, 65, 68]. Results of these models will give us an intuition on how variables are related and where do we have to focus for maximum impact. Microscale tests have to involve experiments related with the analysis of the efficiency of the implemented ecological functions, ecological fitness of modified organisms and long time stability of the system. Basic techniques in biochemistry and molecular biology can be used to measure protein expression level or enzymatic activities. The stability of the incorporated ecological function and the fitness of the modified organisms can be tested in long term growth in bioreactors, with single or mixed populations. Finally, mesoscale experiments, that are the experimental setting that more closely reproduces natural conditions, have to be performed before to releasing SYN organisms.

## Implications of our hypothesis

The four major classes of TMs presented above provide a framework to design synthetic biology alternatives to existing strategies aimed to fight against climate change and its consequences. A main departure from geo-engineering is the fact that designed living machines are by definition capable of self-replication. From an engineering perspective, that implies that the designed biomachines will be capable of making new replicas and thus scale up the problem. The synthetic organisms associated to the TMs act as *ecosystem engineers*, capable of modifying the flows of energy and matter through the ecosystem [27, 28]. This is actually an approach to restoration ecology that is based in the existence of multiple alternative states in complex ecosystems [60, 66].

A major objection to developing this framework in the real natural habitats is the potential for evolving undesirable (or unexpected) traits. This could be labeled as the “Jurassic Park Effect”: even designed systems aimed to population control can eventually escape from genetic firewalls [14]. This is a claim that is supported by the unescapable potential of microbial systems for evolution. However, two important points need to be made. One is that microbes are being constantly dispersed on a global scale without special impact on extant ecosystems. As it occurs with most invaders, they either fail to survive or simply become part of the receptor habitats, where they are over competed by resident species [16]. Secondly, the design principles proposed in this paper consider engineering extant organisms under a cooperation-based framework (thus enhancing mutualistic loops) or taking advantage of human-generated waste that can act as an artificial substrate to support the synthetic organisms. In all cases, a synergetic interaction between design and niche context is at work. Redesigning our ecosystems requires a modification of nature, and dealing with ecosystem complexity face to face [37]. But we should not forget that most biomes in our planet have already been deeply transformed by human activities [18]. Far from what we could expect, they can be diverse, robust and more efficient in terms of nutrient cycling and other components of ecosystem services [41]. Despite the long, sustained and profound anthropogenic impact on many of these novel ecosystems, they can display a richness and resilience that reminds us the potential of nature to reconstruct itself. It is time to decide what we want and what is our role in the future of nature. If we want humans to be part of the biosphere, we need to foresee the future impact of climate change on our planet. Here, a slow response can trigger sudden shifts, perhaps social collapse [58]. Synthetic biology can play a major role, along with all other strategies, to modify ongoing trends. That means redesign nature, but perhaps too to safely exit the Anthropocene with a renewed relationship with ecological systems.

## Reviewers’s comments

### Reviewer’s report I: Eugene V. Koonin, National Center for Biotechnology Information, National Library of Medicine and National Institutes of Health

Solé and colleagues propose to use mutual dependencies between engineered microbes to control and prevent side effects of bioremediation. I find this to be a very clever idea that is likely to have a future. With the increasing potential for genome engineering, this approach is likely to be quite realistic.

I am slightly uncomfortable with the designation of the article a Hypothesis. Although the authors outline experimental approaches that could be used to implement the strategies proposed in the paper, a hypothesis seems to imply more specific, falsifiable predictions. I would rather place this article in Opinion rubric.

Author response: *We understand the view of the reviewer, but looking at the hypothesis section definition, which indicates that the paper needs to be “backed up solely by a survey of previously published results rather than any new evidence” we thought our choice was essentially correct. The field of synthetic biology is still under development, but it clearly enters into an exponential phase, beyond the proof of concept ideas. Although we are not suggesting specific experimental implementations or candidates to the engineering, we do define the specific ecological motifs that seem to be reasonable candidates.*

### Reviewer’s report II: Tom Ellis, Centre for Synthetic Biology and Innovation, Imperial College London and Department of Bioengineering, Imperial College London, London, UK. Nominated by Purificación Lopez-Garcia

The Hypothesis article “Synthetic circuit designs for Earth terraformation” is an excellent and intriguing read proposing feedback-circuit designs that link the growth and function of engineered microbes both to natural microbes and to the factors found in environments where bioremediation is required. Apart from the minor issues given below, my only concern with the article is that is has a radical title but its content is actually quite conservative. The phrase ‘terraforming’ evokes proactive geo-engineering on a huge scale (e.g. polluting Mars with CO2 to build an atmosphere) however, the article quickly regresses to microbe-based bioremediation strategies e.g. for sewage treatment, and this is not particularly new or radical. Could I enthuse the authors to consider adding more far-reaching thoughts to their article? The article seems unnecessarily limited to microbes - why not also consider plants, insects and algae?

Author response: *It is true that the term Terraformation has important implications, particularly in relation with the scales involved. We have tried to present a reasonable account of the potential key ecological motifs consistent with modifications associated to different scales. Because of this, and since we include apparently smaller-scale systems, our proposal might appear closer to bioremediation strategies. However, the cooperation scenario illustrates our point and the potential for large-scale engineering.*

*The mutualistic design can be applied to prevent or even suppress the dynamics of catastrophic shifts in dry land ecosystems. This is a timely problem affecting the future fate of a large fraction of arid and semiarid habitats. These systems are likely to experience rapid transitions towards desert states, threatening the survival of millions of humans and deals with regional scales. By using our designed ecological interactions, it might be possible to displace the system towards safer states but also promote new shifts to alternative ecological regimes. The second and third class of motifs can be seen as closer to bioremediation strategies, but the proposal here is to exploit vast amounts of waste as potential habitats that could be effectively engineered by the synthetic microbes. This mans not only a bioremediation scheme of action. By engineering microbes capable of acting as ecosystem engineers (and not just waste removers) carbon sequestration could be enhanced, transforming these wastelands in effective carbon sinks.*

*It is also true that we have confined ourselves to microbial-based solutions. There are several reasons why we limit our scope to microorganisms. First, because they are fast-growing systems with a large diversity of potential candidates (some already well characterized) and -more importantly- a potential for regional or even global spread. Testing their potential success is likely to be time consuming, since in vitro as well as micro- and mesoscale will be required before any field case scenario if eventually considered. However, other potential candidates should be considered in the future. Engineered plants are especially suitable as additional candidates, and actually the cooperation motifs where the synthetic microbe is a strict mutualist can be understood as engineered plants. Concerning algae and fungi, the soil crust ecosystem offers many alternative paths to be explored.*

The article also seems limited entirely to engineered microbes that propagate by cell growth and division - why not expand this to the potentially more potent (and risky) strategies of genes that propagate via transfer from cells to cells (e.g. via phage, transposons or the recently described CRISPR Gene Drives technology)?

Author response: *The CRISPR-Cas9 technology provides an enormous potential for engineering organisms beyond our seed-and-grow scenario, thus offering the possibility of shortening and improving part of the bioengineering procedures. Because of its potential for engineering genomes, many shortcomings associated to stability and targeting could be overcome. Its potential to prevent spreading of undesirable plagues has already been discussed and the potential benefits for agriculture, environment and health makes it a great candidate for bioengineering. The CRISPR-Cas9 also offers the potential for engineering the two ends of the cooperation motifs (such as a microbe and its host plant) thus creating additional pathways for our proposed intervention. Here we wanted to provide a reasonably simple engineering scenario where both the genetic engineering part and the ecological context can be considered. How this is eventually implemented needs more thinking, but the CRISPR scenario seems an obvious choice, although theoretical efforts towards predicting ecological outcomes are much needed at this point.*

#### Minor issues

A few publications that are highly-relevant for this paper are missing and should be included if possible:

A design case for organisms engineered to protect diversity: www.daisyginsberg.com/work/designing-for-the-sixth-extinction.

Author response: *Added.*

CRISPR Gene Drives: dx.doi.org/10.7554/eLife.03401.

Author response: *Thanks for this important reference. It has been added.*

Bio-containment using ncAAs: dx.doi.org/10.1038/nature14121.

Author response: *Added*.

Bio-containment designs and strategies: dx.doi.org/10.3389/fmicb.2013.00005.

Author response: *Added*.

### Reviewer’s report III: Eörs Szathmary, Department of Plant Systematics, Ecology and Theoretical Biology, Institute of Biology, Eötvös University, Budapest, Hungary, Department of Plant Systematics, Ecology and Theoretical Biology, Research Group of Ecology and Theoretical Biology, Eötvös University and The Hungarian Academy of Sciences, Budapest, Hungary, Parmenides Center for the Conceptual Foundations of Science, Munich/Pullach, Germany

This is an interesting paper with potentially useful ideas in it. Indeed an environmental catastrophe is so threatening that every effort should be made try to save our lives.

The hypothetical constructs suggested by the authors are exciting and I am convinced they should be ventilated to the community. Nevertheless I feel that the proposal is too optimistic, or indeed a bit naive. I am concerned with the release of the engineered organisms. It strikes me that the proposed firewalls are ecological but not evolutionary. I see no guarantee for the lack of escape mutants or recombinants.

Author response: *We believe that the combination of careful design of synthetic organisms, as suggested here, combined with environmental constraints offer a powerful ecological and evolutionary firewall. The potential for (undesirable) evolution is likely to be strongly limited due to the limitations imposed by the metabolic burden associated to the genetic constructs and the harsh conditions imposed by drylands, wastelands and sewage. Similarly, designed organisms capable of surviving attached to specific components in plastic waste will have a hard time to evolve into something else beyond what was planned.*

*We think that the current process of climate change is forcing us into ask ourselves what do we want. We might face irreversible shifts in drylands that will cause major economic and humanitarian disasters. Designing a microorganism that can maintain the system away from the shift (thus buying us time) might be the way out from collapse. Is this going to end up in a constant re-engineering from us? This is also a possible outcome, with humans becoming the ultimate keepers of planet stability.*

The authors suggest bioreactor experiments and modelling for the assessment of the feasibility of the proposed technologies. Yet all these things can only provide partial, but of course not complete information. Bioreactors contain only a small fraction of the biota, and in the long run local species compositions are bound to change by species invasions, virus infections, etc. Let me illustrate the worry by a blunt example. The late Bill Hamilton was concerned by the increasing genetic load of the human population. He reasoned that although genetic screening techniques do and will improve, they will never be able the give a complete assessment of the phenotypic consequences of a particular genotype. Granted, the organisms you have in mind are a lot simpler but ultimately you will know whether your engineered organisms behave well only if you introduce them into real habitats, but of course when there is trouble then it is too late.

Author response: *Ecosystem diversity represents an additional layer of complexity that we are not (directly) considering here. Diversity and the heterogeneous nature of real ecosystems are certainly relevant and need to be considered. They can actually act against the success of designed invaders (particularly the ones proposed here) and some theoretical work will be needed to assess the invasion success of our proposed designs under species-rich conditions. Once again, design principles can also incorporate some of our current knowledge of invasion ecology in order to predict potential outcomes. On the other hand, some controlled field experiments done in semi-arid landscapes and dealing with a single keystone species have shown how important is the impact of a single-species modification. This of course does not pervade predictability but tells us that the population dynamics of Terraformation motifs will need to deal with the concept of keystones in the future.*

I remain sceptical about this suggestion. (Please also consider the serious criticism against the naivety of synthetic biology as such, about which you can now read in various places.) I would suggest that people should keep on working out possible remedies in very different domains in parallel. But first and foremost I wish that great powers in the world should become conscious of their enormous responsibility. Without the latter we might fail miserably (although I also might be hopelessly naive hoping for this). There is now indication that solar systems with planetary distributions hospitable to life might be rather rare, even if planets as such are common. This is a broad anti-Copernican twist. Life on earth may thus be more valuable than we used to think.

Author response: *Indeed, a good dose of both skepticism and careful thinking are very much needed. As Edward O. Wilson pointed out, this is “one planet, one experiment” and might seem too bold to even discuss the possibility of manipulating the biosphere. But once again it is also time to consider all possible outcomes and our way out from future breakpoints in a world that we have already been modifying at an accelerated pace. In this context, we have urgent problems to solve and the potential for using science and common sense to find alternatives. Our approach should be considered as an additional element along other rational strategies including sustainable growth and the enforcement of renewable energy use.*

## Abbreviations

H: Host
SYN: Synthetic organism
WT: Wild type
TM: Terraformig motif
R: Resources
W: Water
